# Cortactin Tyrosine Phosphorylation Promotes Its Deacetylation and Inhibits Cell Spreading

**DOI:** 10.1371/journal.pone.0033662

**Published:** 2012-03-30

**Authors:** Eugenia Meiler, Elvira Nieto-Pelegrín, Narcisa Martinez-Quiles

**Affiliations:** Departamento de Microbiología II, Facultad de Farmacia, Universidad Complutense de Madrid, Madrid, Spain; Hungarian Academy of Sciences, Hungary

## Abstract

**Background:**

Cortactin is a classical Src kinase substrate that participates in actin cytoskeletal dynamics by activating the Arp2/3 complex and interacting with other regulatory proteins, including FAK. Cortactin has various domains that may contribute to the assembly of different protein platforms to achieve process specificity. Though the protein is known to be regulated by post-translational modifications such as phosphorylation and acetylation, how tyrosine phosphorylation regulates cortactin activity is poorly understood. Since the basal level of tyrosine phosphorylation is low, this question must be studied using stimulated cell cultures, which are physiologically relevant but unreliable and difficult to work with. In fact, their unreliability may be the cause of some contradictory findings about the dynamics of tyrosine phosphorylation of cortactin in different processes.

**Methodology/Principal Findings:**

In the present study, we try to overcome these problems by using a Functional Interaction Trap (FIT) system, which involves cotransfecting cells with a kinase (Src) and a target protein (cortactin), both of which are fused to complementary leucine-zipper domains. The FIT system allowed us to control precisely the tyrosine phosphorylation of cortactin and explore its relationship with cortactin acetylation.

**Conclusions/Significance:**

Using this system, we provide definitive evidence that a competition exists between acetylation and tyrosine phosphorylation of cortactin and that phosphorylation inhibits cell spreading. We confirmed the results from the FIT system by examining endogenous cortactin in different cell types. Furthermore, we demonstrate that cell spreading promotes the association of cortactin and FAK and that tyrosine phosphorylation of cortactin disrupts this interaction, which may explain how it inhibits cell spreading.

## Introduction

The actin cytoskeleton remodels to accomplish many cellular processes and therefore undergoes significant changes during cell migration, adhesion, endocytosis and bacterial invasion [1]. The cortactin protein has emerged as an important node in the network regulating the actin cytoskeleton during numerous biological processes [2], [3]. It was originally described as a substrate of Src kinase located primarily at the cell cortex [4]. Almost simultaneously, cortactin was cloned as the product of the *CTTN* gene (formerly *EMS1*), located in chromosomal region 11q13, which is frequently amplified in different human carcinomas [5]. Today, cortactin is considered an oncoprotein and a *bona fide* invadopodial marker [6].

Cortactin is a modular protein that contains an N-terminal acidic (NTA) domain with a _20_DDW_22_ motif that directly binds and activates the Arp2/3 complex. The NTA domain is followed by six and a half amino acid ‘repeats’ that bind to F-actin and define the actin-binding region (ABR) [7]. Since cortactin only weakly activates the Arp2/3 complex *in vitro* [8], it is unclear whether cortactin requires post-translational modifications to be fully active. The ABR is followed by a helical, proline-rich region, followed in turn by a C-terminal Src homology (SH3) domain. Cortactin binds several proteins through its SH3 domain, such as WIP [9] and neural Wiskott-Aldrich syndrome protein (N-WASP) [10], [11].

Cortactin regulation is very complex [12]. Although traditionally studied as a substrate of Src family kinases (SFKs) [4], it can also be phosphorylated by other tyrosine kinases such as Fer [13] and Abl/Arg [14]. The effects of tyrosine phosphorylation on cortactin structure and function remain largely unknown. This phosphorylation was shown to decrease cortactin binding to F-actin [15], and this binding is required for cortactin activation of the Arp2/3 complex [16]. This phosphorylation is also required for inducing bone metastasis of breast cancer cells in nude mice [17], and it appears to be involved in bacterial invasion of cells, such as for the adhesion of enteropathogenic *Escherichia coli* (EPEC) [18]. Protein phosphatase 1B (PTB-1B) dephosphorylates tyrosine 421 in cortactin [19], suggesting reversible regulation. The data seem to indicate that tyrosine phosphorylation of cortactin is tightly controlled, but the details of this regulation are far from clear. Tyrosine phosphorylation-dephosphorylation of cortactin may regulate its ability to form complexes with other proteins [20], [21].

Cortactin is also the target of serine-threonine kinases, including ERK [22] and Pak [23], [24]. In fact, phosphoproteomic analysis has revealed numerous phosphorylation sites, most of which are serines and threonines [25].

Cortactin promotes actin polimerization through two pathways: directly, by activating the Arp2/3 complex; and indirectly, when the SH3 domain binds and activates N-WASP [10]. *In vitro*, cortactin binds and activates N-WASP only when phosphorylated on serines by ERK, whereas phosphorylation by Src at tyrosines 421, 466 and 482 terminates cortactin activation of N-WASP, which suggests that phosphorylation indeed affects cortactin structure. Based on studies by our group [10] and others [22], we proposed a model in which serine/tyrosine phosphorylation controls the accessibility of the SH3 domain of cortactin [10]. This model was subsequently named the ‘S-Y Switch’ model [26], and its most easily testable prediction is that cortactin can be regulated by a conformational change. The structure of unmodified cortactin [27] reveals a closed, globular conformation achieved mainly through interactions between the SH3 domain and ABR region.

Studies with mutant forms of cortactin have been carried out to understand the functional consequences of serine and tyrosine phosphorylation. Indeed it has been proposed that different cortactin phosphoforms have distinct cellular functions: in this proposal, tyrosine-phosphocortactin mainly regulates focal adhesion turnover, whereas serine-phosphocortactin controls actin polimerization [28]. More recently, antibodies specific for phospho-serine have been used to show that serine phosphorylation of cortactin is essential for lamellipodial persistence [29].

Adding another layer of complexity to cortactin regulation, studies have shown that the protein is also regulated by reversible acetylation. The protein can be acetylated by histone acetyltransferase p300/CBP-associated factor (PCAF) and deacetylated mainly by Histone Deacetylase 6 (HDAC6). Acetylated cortactin has a reduced capacity to bind F-actin [30].

Although numerous studies of cortactin have suggested a complex network of regulatory post-translational modifications, they have been unable to indicate definitively how Src-mediated phosphorylation affects cortactin structure and activity, and how this phosphorylation relates to other post-translational modifications. These difficulties may reflect the low basal level of phospho-tyrosine cortactin in most cell types, which makes cell culture-based studies of cortactin challenging. Here we attempt to overcome this problem using the Functional Interaction Trap (FIT) system [31], [32]. The FIT system involves fusing a kinase and a substrate of interest to complementary leucine zippers; cotransfection with the two expression vectors allows for specific and efficient phosphorylation of the substrate.

## Methods

### Cells, reagents and antibodies (Abs)

The following cell lines were obtained from the American Type Culture Collection (ATCC): human epithelial HeLa cells; mouse fibroblasts deficient in Src, Yes, and Fyn kinase (Src^−/−^, Yes^−/−^, Fyn^−/−^; abbreviated SYF); and SYF fibroblasts rescued for Src (Src^+/+^, Yes^−/−^, Fyn^−/−^; abbreviated Rsrc). Wild-type (WT) and HDAC6-deficient MEFs immortalized by p53 gene deletion [33] were obtained from Dr. Tso-Pang Yao (Department of Pharmacology and Cancer Biology, Duke University). Cells were grown in Iscove's modified Dulbecco's medium (IMDM) supplemented with 10% fetal bovine serum (FBS) and antibiotics. The deacetylase inhibitor Trichostatin A (TSA) from *Streptomyces sp* was purchased from Sigma. The selective Src-family kinase inhibitor PP2 was purchased from Calbiochem.

The following commercial Abs were purchased from Millipore: mouse cortactin 4F11 MoAb; mouse Src GD11 MoAb; mouse myc 4A6 MoAb; Platinum phospho-tyrosine (for WB), which is a mixture of two generic phosphotyrosine Abs: PY20 and 4G10; vinculin MoAb; and FAK 2A7 MoAb (for IPs). For immunoprecipitations (IPs), we used phosphotyrosine MoAb and for FAK WB, the Ab from Cell Signaling. Mouse actin C4 MoAb was from MP Biomedicals, and rabbit cortactin MoAb was from Novus Biologicals. The rabbit cortactin polyclonal Ab (Applied Biological Materials) was raised against an unphosphorylated peptide around tyrosine 466. This Ab recognizes both unphosphorylated and tyrosine-phosphorylated cortactin. Rabbit phosphocortactin Y466 polyclonal Abs were obtained from Santa Cruz Biotechnology and from Abcam (data not shown). Myc (A14) Ab was from Santa Cruz. pY421 cortactin Ab was from Abcam. Rabbit Ab against acetyl-cortactin was initially obtained from Dr. Edward Seto (H. Lee Moffitt Cancer Center and Research Institute, Tampa, Florida) and subsequently from Millipore.

IRDye 800CW-labeled goat rabbit and mouse secondary Abs (Fisher Scientific) were used to give green signal. IRDye 680CW-labeled goat rabbit Ab (Fisher Scientific) and Alexa 680-labeled goat mouse Ab (Invitrogen) were used to give red signal. All secondary Abs were purchased at a concentration of 1 mg/ml and used at 1∶5,000 dilution.

### Constructs

All FIT constructs used, including MycCortactin, were a generous donation of Dr. Bruce J. Mayer (Connecticut Health Center, CT, USA). Cortactin with mutations of all three tyrosines that can be phosphorylated by Src (Y421/466/482F, referred to as the 3F mutant) was produced using the QuikChange site-directed mutagenesis kit (Stratagene). Mutations were produced sequentially: first the 421F mutant was generated, and then this was used as template to mutate Y466 and Y482 (primer sequences available upon request). After verifying the sequence, the insert was subcloned into an empty ZipB vector. WT GFP-cortactin and 3F-GFP constructs were previously described [10].

### Cell transfection and Western blotting (WB)

For transfection, plasmid DNAs were purified with endotoxin-free, transfection-grade JetStar 2.0 Midi columns (Genomed) as per the manufacturer's instructions. Cell transfections were carried out using Lipofectamine 2000 (Invitrogen) or Fugene HD transfection reagent (Roche). Briefly, cells were grown to 60–75% confluence for Lipofectamine transfections or to 50–60% confluence for Fugene transfections in 6-well plates using 2 µg of the indicated plasmids per well. Transfections were incubated for approximately 20 h in medium containing 10% FBS but no antibiotics.

WB was carried out on cells from a single well or, when necessary, from a 100-mm plate. Cells were washed once with cold Dulbecco's phosphate-buffered saline (D-PBS) with calcium and magnesium (Invitrogen) and scraped into 300 µl 2× Laemmli buffer. Samples were homogenized by three passages through a syringe with a 25-gauge needle and then centrifuged at 21,000×*g* for 5 min at 4°C. Samples were resolved by 10% SDS-PAGE and transferred to nitrocellulose membranes (Amersham) using a BioRad transfer system. Membranes were blocked for 1 h with Odyssey blocking buffer and incubated overnight with primary Ab in blocking buffer containing 0.1% Tween 20. Membranes were washed 4 times for 5 min with PBS containing 0.1% Tween 20, then incubated for 1 h with the appropriate secondary antibody, and washed as before. Membranes were scanned with the Odyssey infrared system (Lycor, Fisher Scientific) using the red (700 nm) and green (800 nm) channels. When required, membranes were stripped using Odyssey stripping buffer according to the manufacturer's instructions. When significantly different intensities were observed between the two color signals, we performed sequential Ab incubations. After stripping membranes, we incubated them with secondary Ab alone and scanned them to confirm the efficiency of stripping before incubating them with another primary Ab.

Quantification of the bands was performed on the scanned images using the Odyssey Scan band tool. The results were analyzed by the two-tailed Student's t test and displayed graphically using GraphPad Prism software (version 5.0).

### Pervanadate treatment

Pervanadate solution was prepared by mixing 1 mM Na_3_VO_4_ with 1% H_2_O_2_ (both from Sigma), diluting two-fold with IMDM medium and used for 30 min at 37°C and 5% CO_2_.

### Immunoprecipitation (IP) experiments

Cells were grown on 150-mm plates and transfected as described above with 20 µg of each plasmid. After transfection cells were washed once with D-PBS and scraped into 700 µl modified RIPA buffer [50 mM Tris-HCl (pH 7.4), 150 mM NaCl, 15% glycerol, 2 mM EDTA, 0.1% SDS, 1% Triton X-100, 1 mM Na_3_VO_4_, 10 mM NaF, 1 mM PMSF, protease inhibitor cocktail (Amersham), phosphatase inhibitor (PhosSTOP, Roche)]. When indicated, TSA was added to the RIPA buffer at a final concentration of 400 ng/ml to detect cortactin acetylation [30], except in the case of lysates from WT or HDAC6-deficient cells.

Magnetic mouse or protein G Dynabeads (30 µl/IP, Invitrogen) were washed and blocked with PBS containing 0.1% BSA for 10 min, then incubated 1 h with 4 µg Ab per IP. After one wash with PBS-0.1% BSA, the beads were added to 200–300 µl cell lysate and incubated with rotation at 4°C for 4 h. The beads were washed 3 times with the help of a magnet (Invitrogen) and 200 µl lysis buffer diluted 1∶10 in PBS supplemented with TSA, except in the case of lysates from WT or HDAC6-deficient cells, when TSA was omitted. The beads were resuspended in 40 µl 2× Laemmli buffer and processed for SDS-PAGE or frozen at −80°C until further analysis.

### Pull-down (PD) experiments

GST and the GST-cortactin SH3 domain were produced in BL21 *E. coli*, purified and coupled to GSH-beads [10]. The proteins were added to 200 µl cell lysate and incubated for 3 h with tumbling at 4°C. Pull-downs were washed twice with 200 µl lysis buffer diluted 1∶10 in PBS.

### Immunofluorescence microscopy

Cells were fixed for 20 min at room temperature with 4% formalin (Sigma) and permeabilized with 0.1% Triton X-100 for 5 min. After three washes with PBS, cells were blocked with 2% BSA in PBS for 10 min, stained at room temperature (RT) with the appropriate primary Ab for 1 h, washed 3 times with PBS, and finally incubated 1 h with secondary Ab. Actin cytoskeleton was visualized with 1 µg/ml tetramethyl-rhodamine-isothiocyanate (TRITC)-phalloidin (Sigma) or a 1∶25 dilution of Alexa Fluor 350-phalloidin (Invitrogen). The secondary Abs (Invitrogen) were Alexa Fluor 405-labelled mouse (blue), Alexa Fluor 488-labeled mouse and -rabbit (green), and Alexa 568-labeled rabbit (red).

Counting was done using a Nikon Eclipse TE 200-U fluorescence microscope equipped with a Hamamatsu camera. Images were processed with Adobe Photoshop. Confocal microscopy was performed at the Parque Científico de Madrid microscopy facility with a Leica Confocal SP2/DMEIR2, using Leica software (version 2.61).

### Spreading experiments

Cells were transfected for 20 h, trypsinized and washed once with trypsin inhibitor at 0.5 mg/ml (Sigma). For immunofluorescence studies, 2.5×10^5^ cells per time point were replated on 4 coverslips previously treated with 30 µg/ml fibronectin (Calbiochem) in each well of a 6-well plate. After fixation they were processed for immunofluorescence. For FAK pull-down and IP experiments, transfections were carried out in 150-mm plates, and cultures were trypsinized 20 h later. For each condition, 2.5×10^6^ trypsinized cells were kept in suspension or replated on fibronectin-treated 100-mm plates.

## Results

### Efficient tyrosine phosphorylation of cortactin by Src in cells transfected with the FIT system

**Figure 1:**
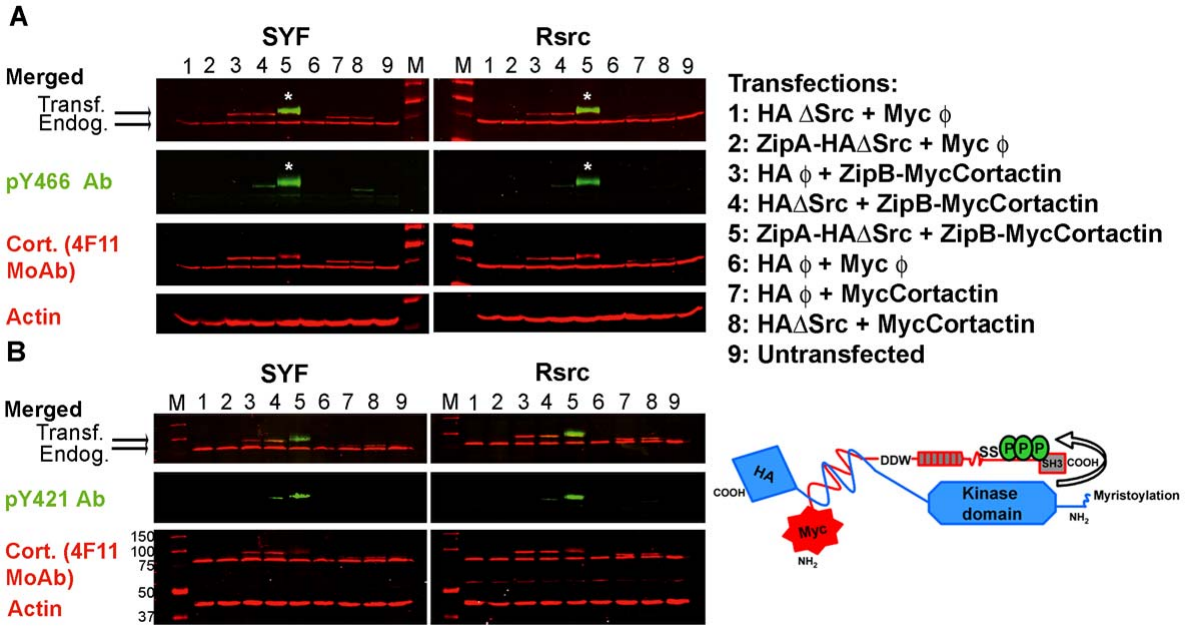
Efficient tyrosine phosphorylation of cortactin by Src in cells using the FIT system. SYF and Rsrc cells were transfected with different combinations of Src and cortactin FIT fusion vectors (lanes 1–8) or left untransfected (lane 9). Cell lysates were blotted for actin as a loading control and with different Abs, then blotted with the respective conjugated secondary antibodies and finally visualized with the Odyssey system. The lysates were blotted with (**A**) pY466 or (**B**) with pY421 cortactin Abs. In both cases, we observed a clear specific phosphorylation band (in green) when ZipA-HA-ΔSrc and ZipB-MycCortactin were cotransfected (transfection 5), and this band superimposes (asterisks) on the cortactin band detected with the 4F11 MoAb (in red). Sizes of the molecular weight markers (denoted M) are shown in kDa. A schematic cartoon of the FIT system is shown.

To specifically phosphorylate cortactin in cells with Src kinase, we used the FIT system (Fig. 1, schematic cartoon). HA-tagged Src kinase lacking the SH2 and SH3 domains was expressed with a C-terminal leucine zipper from the Leucine-ZipA vector (ZipAHA-ΔSrc), while myc-tagged cortactin was expressed with an N-terminal leucine zipper from the Leucine-ZipB vector (ZipBMyc-Cortactin) (Fig. 1, lane 5). As controls, we transfected different vector combinations (Fig. 1A) and left cells untransfected (lane 9). We performed these transfections in SYF fibroblasts, which lack the three SFKs (Src, Yes, Fyn) predominant for that cell type [34]. We also performed these transfections in control cells reconstituted with Src (Rsrc cells).

WB of cell lysates was carried out using the Odyssey two-color infrared scanning system. Src targets tyrosines 421, 466, and 482 of mouse cortactin [35]. We observed strong tyrosine phosphorylation of transfected cortactin using a phospho-specific Ab against tyrosine 466 (pY466 Ab). Simultaneously we detected both endogenous cortactin, migrating at 80–85 kDa, and transfected cortactin using the 4F11 mouse MoAb (Fig. 1A). We next merged the images to show that the phosphorylated band superimposes on the transfected cortactin band, and that the mobility of both bands was slightly lower than that of unphosphorylated cortactin. Actin was detected as a loading control. Similar results were obtained with a different cortactin pY466 Ab (data not shown). Using the FIT system, we found no appreciable differences in the levels of cortactin phosphorylation between SYF and Rsrc cells.

To analyze how efficiently the FIT system generated phosphorylated cortactin, we transfected truncated Src kinase and cortactin without the ZipA or ZipB domains, respectively (Fig. 1A, lines 4 and 8). The results show that cotransfection of Src and cortactin increases tyrosine phosphorylation of cortactin, and that the phosphorylation level is much higher when the leucine zipper domains are used, in agreement with previous studies using the FIT system [32].

We also analyzed the phosphorylation of position 421 using a pY421 Ab (Fig. 1B). The results in Fig. 1A and 1B indicate that the FIT system allows efficient phosphorylation of cortactin on tyrosines 421 and 466. In subsequent experiments, we used the pY466 Ab because it gave a stronger signal than the pY421 Ab.

To specifically detect transfected cortactin, we performed WB with mouse myc 4A6 MoAb and rabbit cortactin MoAb. Fig. S1A shows that transfected cortactin is recognized equally well by both Abs. This was further confirmed by the superposition of bands generated with the 4F11 Ab and the rabbit cortactin MoAb (data not shown). These results show that endogenous and transfected ZipBMyc-Cortactin can be detected by either MoAb, though the signal intensity was greater with the rabbit MoAb.

As a control for the transfections we blotted with antibodies against the HA tag to detect both HA-ΔSrc (Fig. S1B, lanes 1, 4 and 8) and ZipAHA-ΔSrc (lanes 2 and 5). We confirmed the genotype of the SYF and Rsrc cells by blotting with Src MoAb GD11 (Fig. S1B).

### Substrate specificity of Src kinase in the FIT system

To determine whether cortactin is the primary Src substrate phosphorylated in cells transfected with the FIT system, we analyzed the cell lysates by WB using mouse MoAbs against generic phospho-tyrosine (Platinum Ab: 4G10+PY20) (Fig. S2). The same membrane was also incubated with rabbit cortactin MoAb. We observed a strong phospho-tyrosine band that comigrated with transfected cortactin (lane 5, asterisk). When Src kinase is activated, it is phosphorylated on tyrosines, which explains why we observed in lanes 2 and 5 a band of slightly higher molecular weight than actin that corresponds to ZipAHA-ΔSrc. Similarly, we observed Src kinase in the reconstituted Rsrc cells. These results demonstrate that the major phospho-protein in our lysates is transfected ZipBMyc-Cortactin that is tyrosine-phosphorylated by ZipAHA-ΔSrc (lane 5, asterisk).

**Figure 2:**
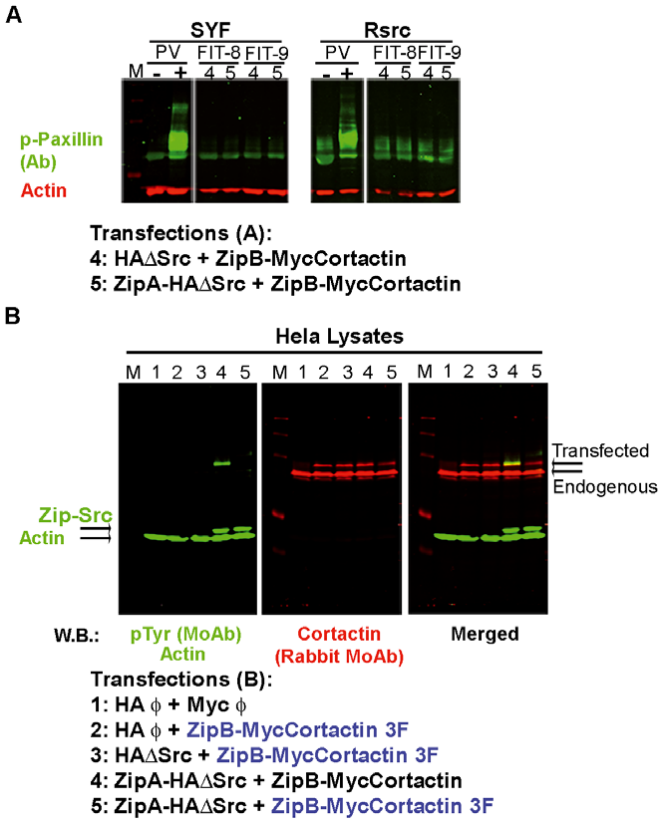
Specificity of tyrosine phosphorylation in the FIT system. (**A**) Detection of the phosphorylation status of paxillin, another Src kinase substrate. SYF and Rsrc cells were transfected with FIT fusion vectors and the most relevant lysates (4 and 5) from two different experiments (FIT 8 and 9) were analyzed by WB with a rabbit Ab against phospho-paxillin (in green) and with a MoAb against actin (in red). As controls, cells were left untreated or treated with pervanadate (PV), a potent phosphatase inhibitor that induces the phosphorylation of paxillin. Rsrc cells showed a higher basal level of phospho-paxillin than did SYF cells, though in both cell lines, this basal level was enhanced by treatment with PV. The FIT system did not increase the basal level of phospho-paxillin. (**B**) Tyrosine phosphorylation of cortactin occurs on the expected tyrosines (Y421, Y466 and Y482). HeLa cell lysates were transfected with ZipA-HA-ΔSrc and ZipB-MycCortactin (lane 4) or with ZipA-HA-ΔSrc and ZipB-MycCortactin with the triple mutation Y421/466/482F (3F) (lane 5). Several control cotransfections were done (lanes 1–3). WB with generic pTyr MoAb demonstrated that only ZipB-Myc WT cortactin, and not the 3F mutant, was phosphorylated (in green). Cortactin was detected with a rabbit MoAb (in red). Actin is shown as a loading control.

As a second control of phosphorylation specificity, we analyzed whether the Src substrate paxillin [36] is phosphorylated by our transfected Src kinase. We performed WB using a phospho-paxillin (p-paxillin) Ab. In Fig. 2A we show the most relevant transfections (lanes 4 and 5) from two FIT experiments (FIT8 and FIT9), after blotting with p-paxillin Ab. As an internal control, we treated both cell types with pervanadate, a generic phosphatase inhibitor that induced a strong signal for p-paxillin. While untreated cell lysates did not show detectable paxillin phosphorylation, lysates of treated cells did. Thus we can conclude that our transfected cells express a basal level of phospho-paxillin.

### Positional specificity of Src kinase in the FIT system

Src phosphorylates tyrosines 421, 466 and 482 in mouse cortactin [35]. After detecting cortactin phosphorylation at positions 421 and 466, we wanted to exclude the possibility of phosphorylation at other tyrosines. For this purpose we used a non-phosphorylatable mutant in which the three major residues targeted by Src kinase were replaced by phenylalanine (3F). We carried out our experiments in HeLa cells because the experiments described above showed similar results in SYF and Rsrc cells, and HeLa cells are easier to handle and widely used.

We cotransfected cells with ZipAHA-ΔSrc and either ZipBMyc-Cortactin or ZipBMyc-Cortactin 3F, and performed WB using Abs against generic phospho-tyrosine and cortactin. Cortactin was phosphorylated on tyrosines, while the 3F mutant was not (Fig. 2B, lanes 4 and 5), indicating that cortactin is phosphorylated specifically on the expected tyrosines.

### Relationship between tyrosine phosphorylation and acetylation of cortactin

Cortactin is acetylated mainly in the cortactin repeat region, and this modification decreases the ability of cortactin to bind F-actin [30]. Because cortactin phosphorylation by Src has a similar effect [15], we wanted to analyze whether a relationship exists between the two post-translational modifications.

**Figure 3:**
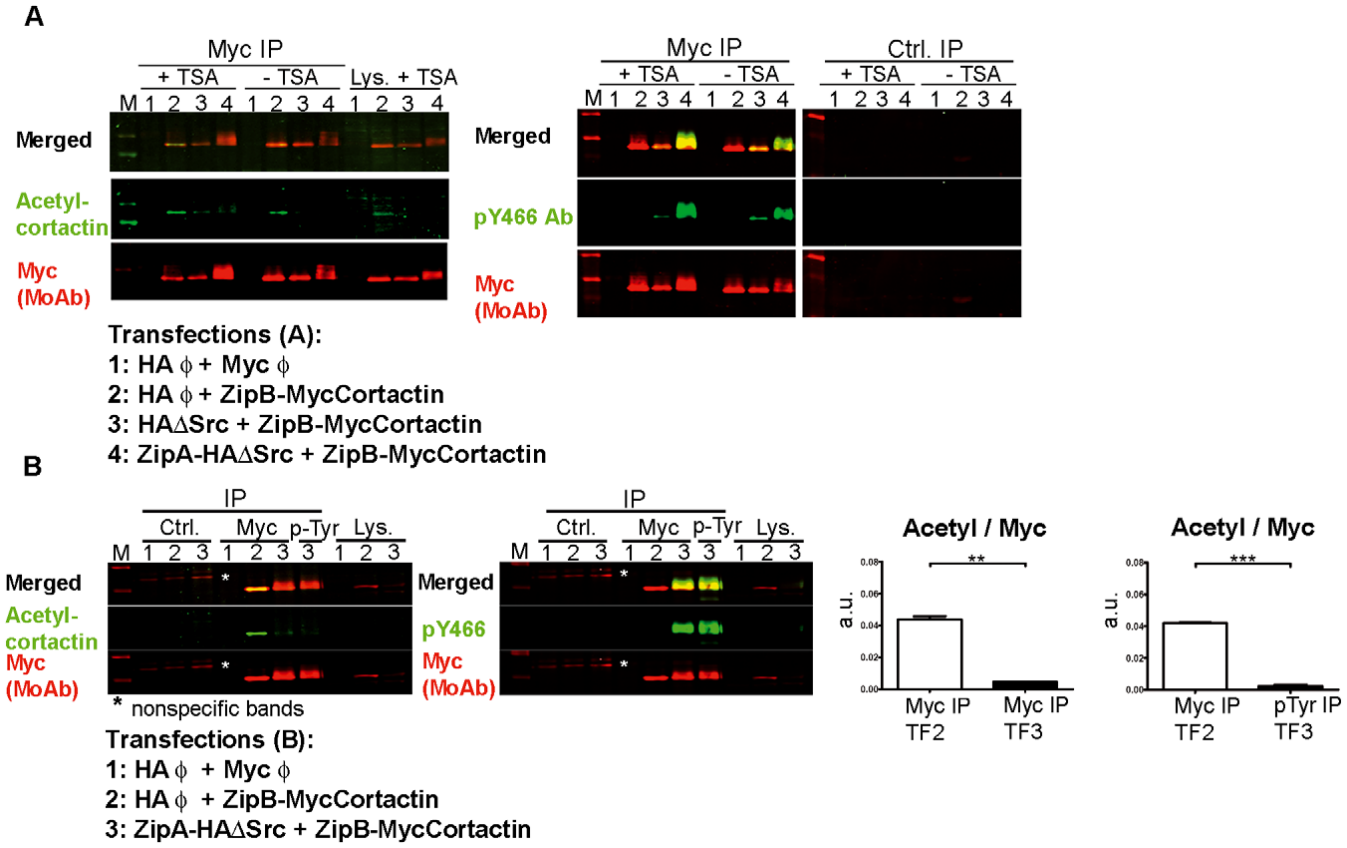
Analysis of acetylation and tyrosine phosphorylation of transfected cortactin. (**A**) Lysates from various transfection combinations (lanes 1–4), treated or not with the deacetylase inhibitor Trichostatin A (TSA), were used to perform IPs using a myc MoAb that were examined by WB first with acetyl-cortactin Ab (in green) and second with myc MoAb (in red). The merge of both images is shown. After the membrane was gently stripped to remove the acetyl signal, it was blotted with pY466 Ab. The isotype control IP (Ctrl.) is also shown. (**B**) TSA-treated cell lysates from various transfection combinations (lanes 1–3) were subjected to parallel IP experiments with the myc MoAb and the generic pTyr MoAb. The IPs were blotted first with acetyl-cortactin Ab, and second with the myc MoAb; then the membranes were stripped and reprobed with pY466 Ab and myc MoAb. The asterisks denote non-specific bands. Quantification of the signals from cortactin immunoprecipitates showed a statistically significant inverse relationship between acetylation and tyrosine phosphorylation signals. a.u.: arbitrary units. *, p<0.05; **, p<0.01.

For this purpose, we decided to use HeLa cells because cortactin acetylation was previously detected in this cell type [30]. Cells were transfected with empty vectors (Fig. 3, lane 1), with ZipBMyc-Cortactin plus HA empty vector (lane 2), or with ZipBMyc-Cortactin together with HA-ΔSrc (lane 3) or ZipAHA-ΔSrc (lane 4). Transfected cells were left untreated or treated with Trichostatin A (TSA), a deacetylase inhibitor previously used to prevent deacetylation of cortactin [30]. WB experiments were performed to confirm tyrosine phosphorylation of cortactin using the pY466 Ab (Fig. S3A). As expected, cortactin was strongly phosphorylated when ZipBMyc-Cortactin was cotransfected with ZipAHA-ΔSrc (lane 4), and the phosphorylation signal was much lower when ZipBMyc-Cortactin was cotransfected with HA-ΔSrc (lane 3).

To analyze whether phospho-cortactin was simultaneously acetylated, we performed IPs of transfected cortactin using a myc MoAb, followed by WB with an Ab specific for acetyl-cortactin (Fig. 3A). The results show that cortactin was efficiently immunoprecipitated in all samples, while it was undetectable in the isotype control IP (Fig. 3A, right panels). Acetyl-cortactin was nearly undetectable in the sample in which cortactin was strongly phosphorylated (lane 4), whereas it was clearly detectable when cortactin was not phosphorylated (lane 2). Treating cells with TSA increased the apparent level of acetyl-cortactin, suggesting that it prevents the deacetylation of cortactin as previously described [30].

In addition to checking cortactin phosphorylation in the lysates used to perform the IPs (Fig. S3A), we wanted to check the phosphorylation status in the immunoprecipitates (Fig. 3A). The membrane was gently stripped until the green acetyl signal was lost and then reprobed with pY466 Ab and myc MoAb. As expected, the myc immunoprecipitates showed cortactin phosphorylation mainly when ZipAHA-ΔSrc and ZipBMyc-Cortactin were cotransfected (lane 4). When transfected alone, ZipBMyc-Cortactin was not detectably phosphorylated, yet it presented a strong acetylation signal (Fig. 3A, lane 2). The results suggest that acetylation and phosphorylation of cortactin occur antagonistically.

To confirm these results separate IPs were carried out in parallel with myc MoAb and generic phospho-tyrosine MoAb (pTyr MoAb) (Fig. 3B). To simplify the experiment we used only TSA-treated cells and the most relevant vector combinations: empty vectors (lane 1), empty HA-vector and ZypBMyc-Cortactin (lane 2), and ZipAHA-ΔSrc and ZipBMyc-Cortactin (lane 3). IP with pTyr MoAb was performed only in the cotransfection of ZipAHA-ΔSrc and ZipBMyc-Cortactin, where cortactin should be phosphorylated. Again, we observed that tyrosine-phosphorylated cortactin was not acetylated and *vice versa*. Thus no signal for acetylation was detected in the phospho-tyrosine IP, which pulled down only tyrosine-phosphorylated cortactin. In contrast, a very faint acetylation signal was detected in the myc IP, which pulled down primarily phosphorylated cortactin but also a small fraction of unphosphorylated protein. We found a statistically significant difference in acetylation signal between unphosphorylated cortactin (transfection 2) and tyrosine-phosphorylated protein (transfection 3; Fig. 3B).

To verify these results with tyrosine-phosphocortactin by a different approach we performed IPs with the pY466 cortactin Ab (Fig. S3B) using the same vector combinations as above (Fig. 3B). The immunoprecipitates and lysates were blotted with cortactin 4F11 MoAb, which detects both transfected and endogenous cortactin in lysates. Tyrosine-phosphorylated cortactin was efficiently immunoprecipitated when ZipAHA-ΔSrc and ZipBMyc-Cortactin were cotransfected (lane 3). WB with acetyl-cortactin Ab revealed that pY466 immunoprecipitates did not contain acetylated cortactin, which is consistent with previous results (Fig. 3A, B). These results indicate that the two modifications did not occur simultaneously, suggesting that a competition exists between phosphorylation and acetylation of cortactin.

**Figure 4:**
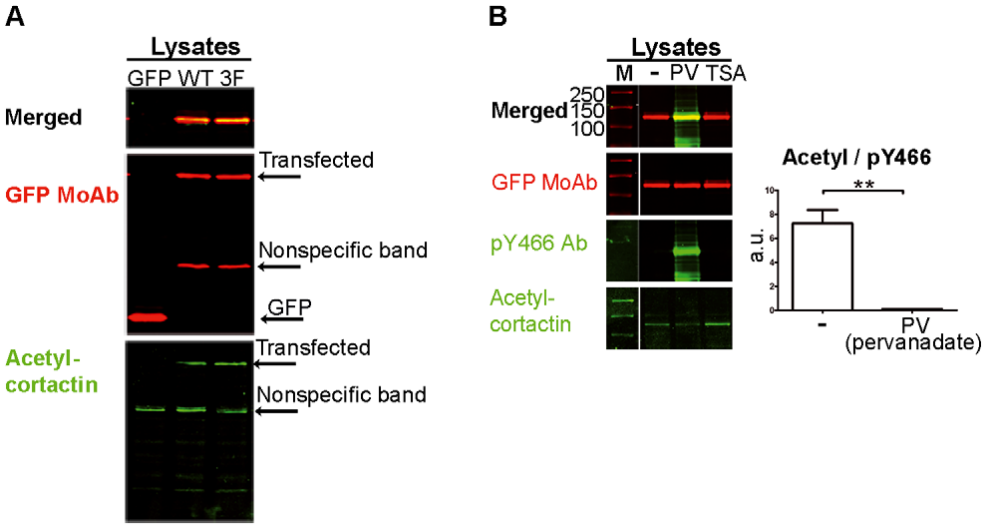
Analysis of acetylation and tyrosine phosphorylation of transfected GFP-cortactin. (**A**) Tyrosine phosphorylation of cortactin is not required for acetylation of the protein. HeLa cells were transfected with vectors encoding GFP fused with WT cortactin or the Y421/466/482F non-phosphorylatable cortactin mutant (3F). Lysates were blotted with acetyl-cortactin Ab and GFP MoAb. Transfected cortactin was acetylated and no statistically significant difference was found in acetylation level between WT and 3F transfectants (data not shown). (**B**) Tyrosine phosphorylation of cortactin decreases acetylation of the protein. HeLa cells were transfected with a vector encoding GFP fused with WT cortactin. Transfectants were left untreated (-) or treated with pervanadate (PV), a generic phosphatase inhibitor, or with Thrichostatin A (TSA), a deacetylase inhibitor. Lysates were blotted with acetyl-cortactin Ab and with GFP MoAb. After stripping, the membrane was incubated with pY466 cortactin, which was merged with the GFP cortactin signal. The ratio of acetyl:pY466 cortactin is shown for untreated (-) and PV-treated cells. a.u.: arbitrary units. **, p<0.01.

To exclude any non-specific effects due to the fusion tag and to further characterize how these two post-translational modifications relate to each other, we performed transfections using GFP-tagged cortactin constructs (Fig. 4). We transfected HeLa cells with empty vector and a vector encoding GFP-WT cortactin or GFP-3F cortactin, and we blotted the lysates with acetyl-cortactin Ab. We observed that WT and GFP-3F cortactin were acetylated (Fig. 4A) and found no statistically significant difference in acetylation level between the two constructs (data not shown). This indicates that phosphorylation of cortactin at positions 421, 466 and 482 is not required for cortactin acetylation.

We next examined the effects of pervanadate (PV) and TSA on GFP-cortactin; these compounds induce phosphorylation and acetylation, respectively (Fig. 4B). Lysates of cells transfected with GFP-cortactin were left untreated or treated with PV or TSA, and subjected to WB with the acetyl-cortactin Ab first, followed by gentle stripping and then reprobing with the pY466 cortactin Ab. As a transfection control, lysates were also blotted with GFP MoAb. Treating lysates with TSA increased the amount of acetyl-cortactin over basal levels. However, we cannot determine whether it simultaneously decreased the level of phospho-cortactin because the basal level of the phospho-protein is undetectable. In contrast, PV strongly induced tyrosine phosphorylation of cortactin, and this was accompanied by a decrease in the level of acetyl-cortactin, such that the ratio of the two forms of cortactin differed significantly from basal conditions (Fig. 4B). These results indicate that induction of tyrosine phosphorylation of cortactin decreases its acetylation.

### Analysis of the acetylation and phosphorylation status of endogenous cortactin

**Figure 5:**
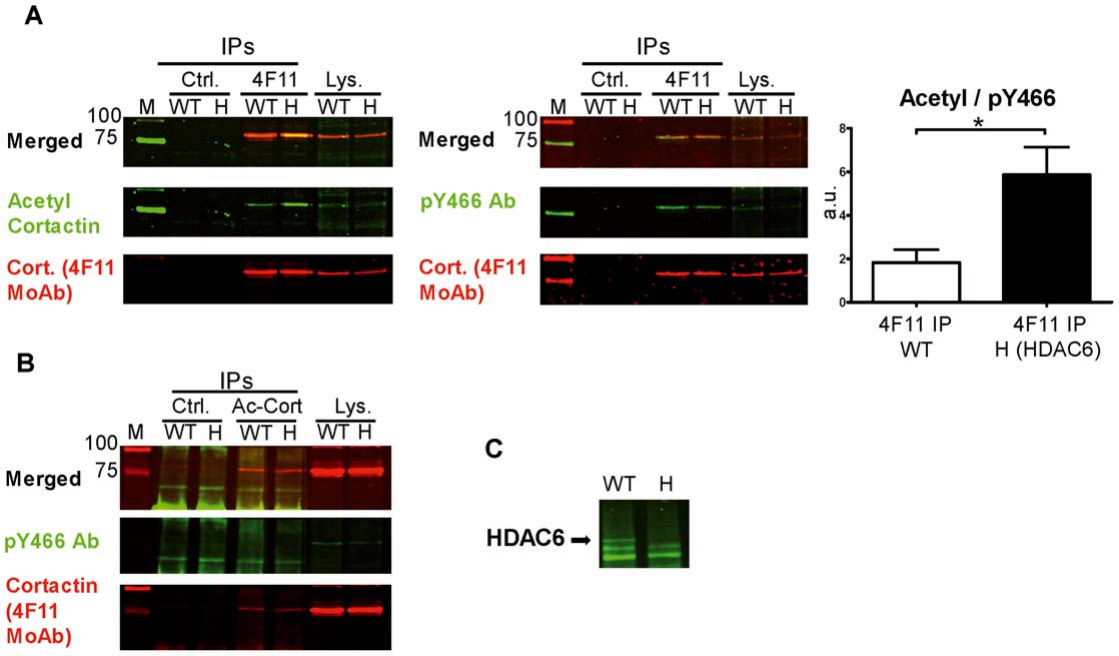
Analysis of acetylation and tyrosine phosphorylation of endogenous cortactin in WT and HDAC6-deficient MEFs. (**A**) Isotype control (Ctrl.) and 4F11 immunoprecipitates from cell lysates of WT and HDAC6-deficient MEFs (H) were blotted first with acetyl-cortactin Ab (in green) and second with the 4F11 cortactin MoAb (in red). The merge of both images is shown. After gentle stripping to remove the acetyl signal, the membrane was blotted with pY466 Ab and 4F11 MoAb. Quantification and statistical analysis of three independent 4F11 immunoprecipitates and the ratio of acetyl:pY466 cortactin signals are shown. a.u.: arbitrary units. *, p<0.05. (**B**) Immunoprecipitates obtained with acetyl-cortactin Ab were blotted with pY466 Ab and 4F11. The phosphorylation signal did not coincide with acetylated cortactin. (**C**) Blotting of WT and HDAC6-deficient cell lysates with HDAC6 Ab is shown as a control of cell phenotype.

**Figure 6:**
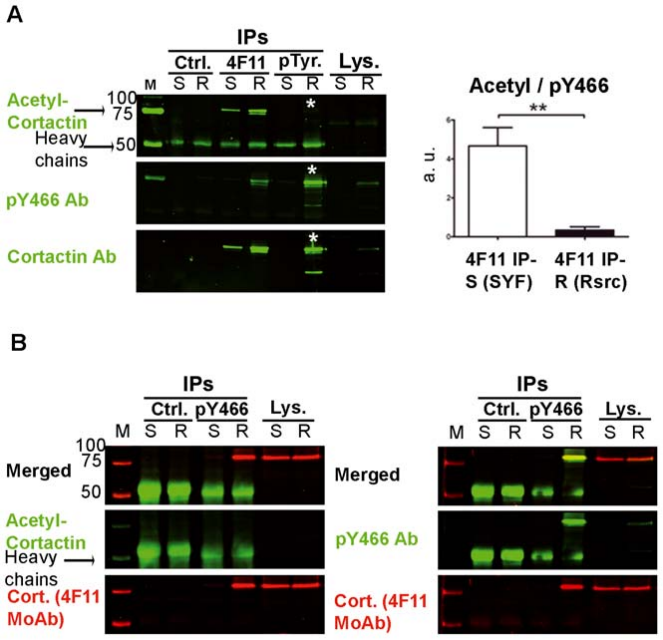
Analysis of acetylation and tyrosine phosphorylation of endogenous cortactin in SYF and Rsrc MEFs. Cell lysates of SYF and Rsrc MEFs were subjected to IPs using (**A**) isotype control Ab (Ctrl), 4F11 MoAb and generic phospho-tyrosine (pTyr) MoAb. These IPs were performed in parallel by probing first with acetyl-cortactin Ab (in green), after a gentle stripping, with pY466 and at last, the membrane was stripped and reprobed with cortactin Ab. Statistical analysis of the ratio of acetyl:pY466 cortactin signals is shown for 4F11 immunoprecipitates. a.u.: arbitrary units. **, p<0.01. Asterisks denote evidence that pTyr immunoprecipitates from Rsrc cell lysates contain phospho-cortactin but not acetyl-cortactin. (**B**) IPs with pY466 and isotype control Abs were probed with acetyl-cortactin Ab and 4F11 cortactin MoAb, and reprobed, after gentle stripping, with pY466 Ab and 4F11 MoAb.

The experiments described so far established that acetylation and phosphorylation of transfected cortactin are mutually exclusive events. We next analyzed whether the same relationship holds for endogenous cortactin (Figs. 5, 6). First we performed experiments in WT and HDAC6-deficient mouse embryonic fibroblasts (MEFs), because HDAC6 is the major cortactin deacetylase in cells [30]. IPs using 4F11 MoAb were blotted with acetyl-cortactin Ab, then the membranes were stripped and blotted with pY466 Ab (Fig. 5A). IPs from HDAC6-deficient cell lysates using 4F11 MoAb showed a significantly higher basal level of acetylated cortactin than did IPs from WT cell lysates. In addition, the ratio of the acetyl:pY466 signals was significantly higher in the HDAC6-deficient cells. These results indicate that the lack of HDAC6 deacetylase significantly increases the acetyl:pY466 cortactin ratio and that HDAC6-deficient cells are a valuable reagent to characterize how acetylation and tyrosine phosphorylation of cortactin relate to each other. Consequently, we performed cortactin IPs using the acetyl-cortactin Ab and blotted them with pY466 Ab and 4F11 MoAb. No signal was detected by pY466 Ab in the immunoprecipitates in which cortactin was detectable with the 4F11 MoAb (Fig. 5B). To confirm this result, acetyl-cortactin immunoprecipitates were analyzed using generic pTyr mouse MoAb or cortactin rabbit MoAb on separate membranes. Similar results were obtained (Fig. S4). We confirmed the HDAC6-deficient phenotype of the MEFs by WB with HDAC6 Ab (Fig. 5C). These results show that endogenous acetylated cortactin is not tyrosine-phosphorylated in WT or HDAC6-deficient cells.

We next examined cortactin acetylation in SYF and Rsrc MEFs, because they represent cell types with different levels of tyrosine-phosphorylated cortactin (Fig. 6). Separate IPs were carried out in parallel using 4F11 and pTyr MoAb and blotted first with rabbit acetyl-cortactin Ab. Then the blot was stripped and reprobed with pY466 cortactin Ab. To detect immunoprecipitated cortactin the membrane was stripped again and reprobed with a cortactin Ab (Fig. 6A). We detected acetyl-cortactin in immunoprecipitates prepared from SYF and Rsrc cell lysates using cortactin 4F11 MoAb. Parallel IPs performed with the pTyr MoAb showed that endogenous tyrosine-phosphorylated cortactin was present only in Rsrc cell lysates, as expected. Furthermore, this cortactin fraction was not acetylated (see asterisks, Fig. 6A). We quantified three independent experiments and found that 4F11 immunoprecipitates from SYF and Rsrc cells differed significantly in the ratio of acetyl:pY466 cortactin (Fig. 6A). These results indicate that when most of the immunoprecipitated cortactin is tyrosine-phosphorylated, then is not concomitantly acetylated.

To confirm these results, we performed IPs from SYF and Rsrc cell lysates with pY466 cortactin Ab (Fig. 6B). The immunoprecipitates were blotted with acetyl-cortactin Ab and cortactin 4F11 MoAb, gently stripped, and then reprobed with pY466 cortactin antibody (Fig. 6B). As detected in Fig. 6A, tyrosine-phosphorylated cortactin was immunoprecipitated only from Rsrc cell lysates. More importantly, cortactin phosphorylated on Y466 was not acetylated (Fig. 6B). Again as detected in Fig. 6A, when cortactin was immunoprecipitated with a generic antibody such as 4F11 MoAb, the immunoprecipitates contained both acetylated and tyrosine-phosphorylated cortactin. On the contrary, when cortactin was immunoprecipitated with a generic phospho-tyrosine MoAb or a specific pY466-cortactin Ab, only tyrosine-phosphorylated cortactin was immunoprecipitated and it was not acetylated. Together these results demonstrate that the majority of endogenous cortactin is acetylated or tyrosine-phosphorylated, consistent with the results obtained with transfected cortactin.

### Analysis of cortactin acetylation and tyrosine phosphorylation during cell spreading

We performed FIT transfections of SYF and Rsrc cells to visualize the location of nonphosphorylated cortactin (transfection 2) and phosphorylated protein (transfection 3). As negative controls, we left cells untransfected and we transfected them with empty vectors (Fig. S5 and data not shown). We visualized cell morphology with TRICT-phalloidin (Fig. S5). We also examined high-magnification images to study cell morphology in detail. We detected cortactin expression using myc MoAb and cortactin phosphorylation using pY466 Ab. As in the IPs (Fig. 6), the level of endogenous cortactin that was tyrosine-phosphorylated in SYF cells under our experimental conditions was nearly undetectable; the level of phosphorylation was similar to that observed in cells transfected only with cortactin (transfection 2, TF2). Cotransfection of cortactin and Src kinase in the FIT system (TF3) increased the level of phospho-cortactin in cells, and this level was easier to observe in SYF cells because of their null background level. In SYF cells, cortactin localized to the cell periphery and around the nucleus, as previously described for endogenous cortactin [7].

In contrast to SYF cells, untransfected Rsrc cells showed, as expected, a detectable level of tyrosine-phosphorylated cortactin (data not shown), as did Rsrc cells transfected in the TF2 experiment. Some cells showed clusters of actin and phospho-cortactin (arrows, Fig. S5). Images from TF3 showed that Rsrc cells were somewhat more retracted and detached than their TF2 counterparts. These results demonstrate that transfected and endogenous tyrosine-phosphorylated cortactin show similar localization, and suggest a role for phospho-cortactin in cell adhesion.

**Figure 7:**
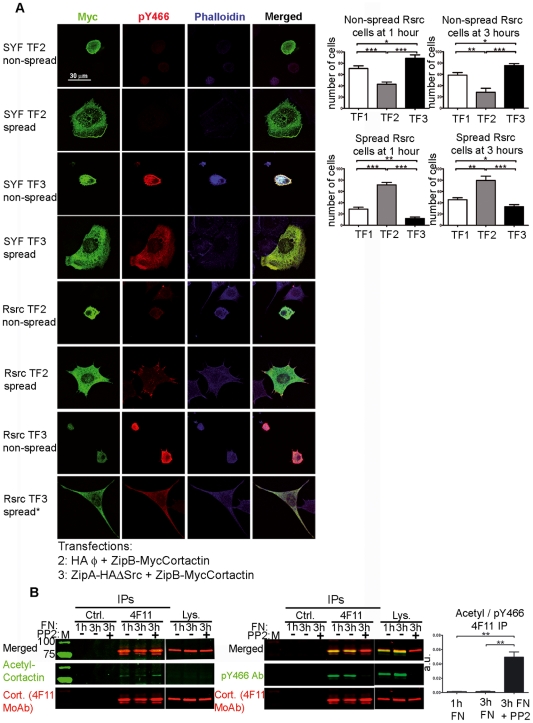
Tyrosine phosphorylation of cortactin affects cell spreading. (**A**) SYF and Rsrc cells were transfected for 20 h with empty vectors (not shown), ZipB-MycCortactin and empty vector (TF2), or ZipB-MycCortactin and ZipA-HAΔSrc (TF3). Cells were then trypsinized, replated on fibronectin-treated coverslips, and fixed at 1 and 3 h. Pictures were taken in a confocal microscope at 600× magnification. Immunofluorescence staining was done using myc MoAb (in green), pY466 cortactin Ab (in red) and Alexa Fluor 350-phalloidin (in blue). For each experimental condition, a representative image of a non-spread and spread cell is shown. * Denotes that spreading of Rsrc cells is incomplete. Images were merged using Leica software. Scale bars are shown. A total of 100 transfected cells were quantified and classified into two categories: spread or non-spread. Statistical analysis from 7 independent experiments at 1 and 3 h after replating Rsrc cells is shown for tranfections TF1 (empty vectors), TF2 (cortactin) and TF3 (phosphorylated cortactin). *, p<0.05; **, p<0.01; ***, p<0.001. (**B**) Inhibition of cortactin phosphorylation increases its acetylation during cell spreading. Rsrc cells were replated on fibronectin (FN)-coated coverslips and allowed to spread for 1 or 3 h. A third plate was allowed to spread for 1 h and then treated with PP2 for 2 h. The lysates were subjected to IPs using isotype control (Ctrl.) MoAb or 4F11 MoAb and were blotted first with acetyl-cortactin Ab and second with anti 4F11 MoAb. After gentle stripping, the membrane was incubated with pY466 cortactin Ab and 4F11 MoAb. Quantification of the ratio of acetyl:pY466 cortactin signals indicated a significantly higher ratio after PP2 treatment. a.u.: arbitrary units. **, p<0.01.

To test this hypothesis, we examined the spreading of transfected SYF and Rsrc cells on fibronectin (Fig. 7). Fig. 7A shows representative spread and non-spread SYF and Rsrc cells transfected with ZipB-MycCortactin and empty HA vector (TF2) or ZipB-MycCortactin and ZipAHA-ΔSrc (TF3). We counted 100 transfected cells and classified then as spread or non-spread for two different time points under three transfection conditions [TF1 (empty vectors), TF2 (cortactin) and TF3 (tyrosine-phosphorylated cortactin)]. In TF3 we counted only transfected cells that also showed significant phosphorylation signal. Quantification of the results showed that at 1 h after replating, cells overexpressing cortactin (TF2) showed significantly more cell spreading than did TF1 or TF3 cells. More importantly, expression of tyrosine-phosphorylated cortactin (TF3) significantly inhibited cell spreading compared to TF1 or cortactin-transfected cells (TF2). This pattern of spreading was also observed at 3 h after replating. Analysis of cells that did not spread confirmed that cortactin favors spreading, while tyrosine-phosphorylated cortactin inhibits it. Indeed, at 6 and 18 h after replating, most Rsrc cells in TF3 were detached (data not shown). Similar results were found in SYF cells, although the differences among the three transfection conditions did not reach statistical significance. These results suggest that cortactin expression favors cell spreading, while cortactin phosphorylation counteracts this effect (Fig. 7A).

To further understand the role of tyrosine phosphorylation of cortactin and explore how it relates to cortactin acetylation during cell spreading, we analyzed the spreading of Rsrc cells on fibronectin in the presence and absence of PP2, a widely used Src family kinase inhibitor (Fig. 7B). Untreated cells were plated and allowed to spread for 1 and 3 h. Cells on a third plate were allowed to spread for 1 h and then they were cultured for 2 h in the medium containing 10 µM PP2. Cell lysates were subjected to IPs with cortactin 4F11 MoAb or isotype control Ab. The membranes were first blotted with acetyl-cortactin Ab, the acetyl signal was stripped, and then the membranes were reprobed with pY466 cortactin Ab and 4F11 cortactin MoAb. We observed that PP2 treatment nearly abolished cortactin tyrosine phosphorylation (right panel IPs, Fig. 7B) and increased the intensity of the acetyl-cortactin signal (left panel IPs). Quantification of three independent experiments showed that treatments with PP2 significantly increased the ratio of acetyl:pY466 cortactin. These results indicate that inhibition of tyrosine phosphorylation of cortactin during cell spreading induces cortactin acetylation.

**Figure 8:**
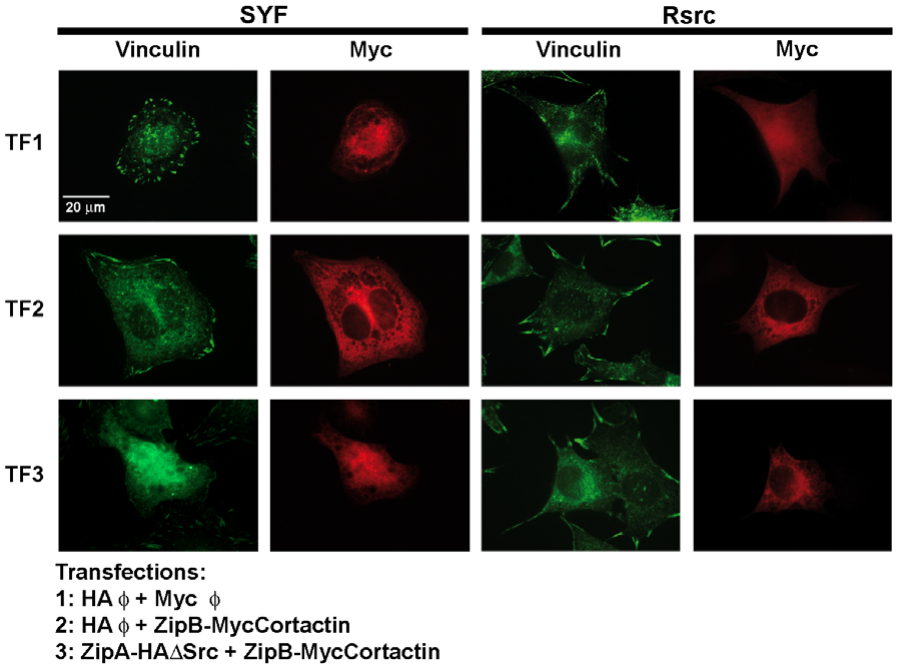
Tyrosine phosphorylation of cortactin affects focal adhesion formation: staining for vinculin. SYF and Rsrc cells were transfected with empty vectors, with ZipB-MycCortactin and empty vector (TF2) or with ZipB-MycCortactin and ZipA-HAΔSrc (TF3). Cells were fixed and visualized by immunofluorescence using vinculin MoAb (in green) and myc Ab (in red). Photographs were taken using a Nikon Eclipse TE 200-U fluorescence microscope equipped with a Hamamatsu camera. Images were processed with Adobe Photoshop. A scale bar is shown.

To characterize how cell spreading is altered by tyrosine phosphorylation of cortactin, we stained focal adhesions using vinculin as a marker (Fig. 8). We performed FIT transfections of SYF and Rsrc cells as before and allowed them to spread on FN for 3 hours (Fig. 8). We visualized focal adhesions by immunofluorescence staining with vinculin MoAb and detected expressed protein using myc Ab in cells transfected with empty vectors (TF1) or in cells overexpressing unphosphorylated cortactin (TF2) or tyrosine-phosphorylated cortactin (TF3). We observed that cells overexpressing tyrosine-phosphorylated cortactin (TF3) showed markedly fewer focal adhesions than under the TF1 and TF2 conditions, and these differences were more apparent in Rsrc cells (Fig. 8) than in SYF cells. Many cells in TF3 had an elongated morphology, in agreement with previous observations (Fig. 7).

### Cell spreading induces the interaction of cortactin with focal adhesion kinase (FAK), and this interaction is lost upon tyrosine phosphorylation of cortactin

To understand how tyrosine phosphorylation of cortactin may affect the formation of focal adhesions, we focused on a recently described cortactin partner, focal adhesion kinase (FAK) [37], which plays important roles in focal adhesion dynamics [38].

**Figure 9:**
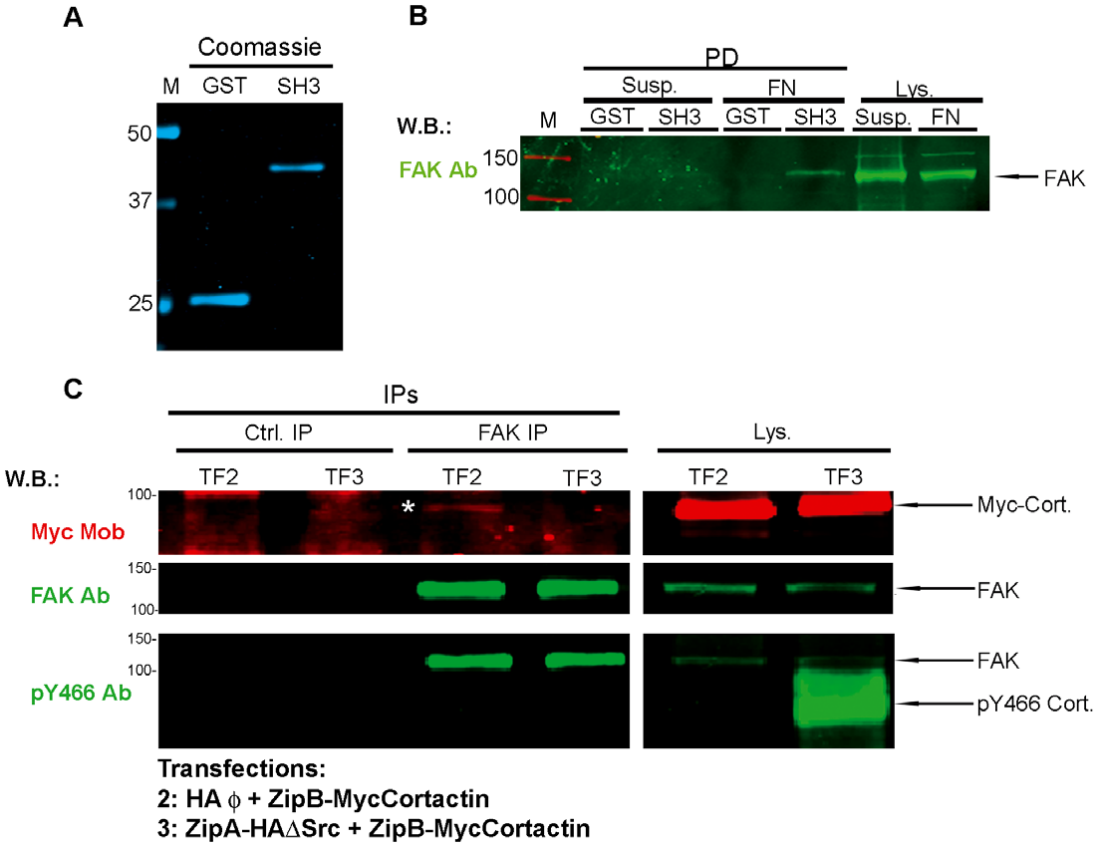
Tyrosine phosphorylation of cortactin terminates its interaction with focal adhesion kinase (FAK) during cell spreading. (**A**) Coomassie staining of purified GST and GST-cortactin SH3 domain was scanned in the Odyssey system. (**B**) HeLa cells were detached with trypsin-EDTA, washed with trypsin inhibitor and kept in suspension (susp.) or allowed to spread for 3 h on fibronectin (FN)-treated 100-mm plates. RIPA cell lysates were used for pull-down experiments with GST or GST-SH3, which were analyzed by SDS-PAGE and WB with focal adhesion kinase (FAK) Ab, followed by labeling with a 800CW-conjugated goat rabbit Ab. (**C**) HeLa cells were transfected with ZipB-MycCortactin and empty vector (TF2) or with ZipB-MycCortactin and ZipA-HAΔSrc (TF3). After 20 h cells were detached with trypsin-EDTA, washed with trypsin inhibitor and allowed to spread on FN-coated 100-mm plates for 3 h. Cell lysates were subjected to immunoprecipitation with FAK MoAb. The immunoprecipitates were subjected to WB and probed in three steps: (1) with myc Ab to detect transfected cortactin, followed by a 680CW-labeled goat mouseAb (red); (2) with FAK Ab, followed by a 800CW-labeled goat rabbit Ab (green); and (3) with pY466 cortactin Ab, followed by a 800CW-labeled goat rabbit Ab. Transfected cortactin was immunoprecipitated by FAK (asterisk) only when the protein was not tyrosine-phosphorylated.

We examined the spreading of HeLa cells on fibronectin, and in parallel left them in suspension as a negative control (Fig. 9). Since previous work has shown that WT cortactin interacts with FAK, while cortactin lacking the SH3 domain does not [37], we performed pull-down experiments on the lysates using recombinant purified cortactin SH3 domain fused to GST (GST-SH3) or GST alone as a negative control (Fig. 9A). We found that cortactin SH3 domain was able to pull down much more FAK from the lysates of spread cells than from lysates of suspended cells. Similar results where obtained with Rsrc lysates (data not shown). These results indicate that focal adhesion formation during cell spreading induces cortactin-FAK association (Fig. 9B).

To confirm the cortactin-FAK interaction during cell spreading and to determine whether it is affected by tyrosine phosphorylation of cortactin, we performed IPs from cell lysates overexpressing cortactin (TF2) or tyrosine-phosphorylated cortactin (TF3) (Fig. 9C). We immunoprecipitated FAK using a FAK mouse MoAb. The immunoprecitates were blotted sequentially, first with myc Ab and secondly with FAK Ab, and lastly with pY466 cortactin Ab. Interestingly, we observed that FAK immunoprecipitated cortactin but not tyrosine-phosphorylated cortactin (see asterisk, Fig. 9C). This result implies that FAK is associated with cortactin, but not when it is tyrosine-phosphorylated.

## Discussion

Cortactin phosphorylation is predicted to have important physiological consequences [26] that are not yet fully understood. Although cortactin is a classical Src kinase substrate, the functional consequences of its tyrosine phosphorylation remain unclear. The low basal level of tyrosine-phosphorylated cortactin, and poor reproducibility of results when stimulating cell cultures with growth factors such as EGF and PDGF, have made it challenging to understand how tyrosine phosphorylation regulates cortactin activity. To avoid these problems, we used the FIT system to study the effect of Src-mediated phosphorylation of cortactin in cells. In this system, a leucine zipper motif, consisting of a pair of complementary amphipathic helices [39], is added to both Src and its substrate, in this case cortactin.

Src kinase targets tyrosines 421, 466 and 482 of murine cortactin [35]. Src family kinases (SFKs) are composed of separable modules that include SH2 and SH3 domains [34]. Different systems have been used to study SFK substrates and signal transduction pathways. The hemopoietic cell Src kinase (Hck) was reengineered by substituting the SH2 and SH3 domains with a PDZ domain to alter the kinase's substrate specificity [40]. In another study, a temperature-sensitive vSrc mutant was found to increase tyrosine phosphorylation of cortactin [41]. The FIT system has been used successfully to force efficient phosphorylation of desired substrates in cells [31], including Src-mediated phosphorylation of paxillin, p130Cas and cortactin [32].

In the present study, we set up the FIT system and simultaneously detected levels of total cortactin and tyrosine-phosphorylated protein, both transfected and endogenous, using two commercial Abs against phospho-Y466 and the 4F11 MoAb (Fig. 1A). Transfected cortactin was also detected using a rabbit cortactin MoAb and a MoAb to recognize the myc tag on our cortactin constructs. Western blots were visualized with the Odyssey scanning system, which allowed unambiguous double labeling of cortactin and phospho-cortactin. We performed these experiments in SYF and Rsrc fibroblasts to exclude any contribution from endogenous Src kinases. When cells were cotransfected with Src and cortactin, both fused to leucine zipper interaction motifs, we observed a strong phosphorylation signal at positions 421 and 466 that superimposed on the transfected cortactin band (Fig. 1A, B).

We next performed various analyses to determine the phosphorylation specificity of our FIT system. We determined that cortactin is the major phosphoprotein in our samples, based on experiments using two generic phospho-tyrosine MoAbs (Fig. S2). We also showed that our FIT system is specific to cortactin: the transfections did not affect the phosphorylation status of paxillin, a known Src substrate (Fig. 2A). Finally, using the non-phosphorylatable triple cortactin mutant Y421/466/482F (3F), we verified that Src-mediated phosphorylation of cortactin occurs at the expected tyrosines (Fig. 2B).

Like phosphorylation, acetylation regulates numerous cellular functions. In fact, many proteins related to cytoskeletal dynamics are regulated by acetylation, such as Arp2/3, tubulin, cofilin and coronin [42]. Cortactin is regulated by reversible acetylation that occurs mainly in the ABR of the protein [30], and this acetylation was recently confirmed by “acetylome” analysis [42]. Acetylation of lysines in the ABR was shown to reduce binding to F-actin, which inhibits cell migration [30].

Like cortactin acetylation, Src-mediated phosphorylation of cortactin decreases its binding to F-actin [15]. This binding is required to activate the Arp2/3 complex [16]. We hypothesized that the two modifications are interrelated since they have similar effects on cortactin function, and we explored this idea in the present study. Using the FIT system, we overexpressed phosphorylated or unphosphorylated cortactin in cells, immunoprecipitated the protein, and performed WB experiments with Abs against acetyl-cortactin and pY466-cortactin (Fig. 3). To be sure of our results, we performed the IPs using a myc MoAb, and then again using a generic phospho-tyrosine MoAb (Fig. 3A, B) and pY466 cortactin Ab (Fig. S3). The first set of IPs brought down phosphorylated and unphosphorylated cortactin, whereas the second set brought down only phosphorylated cortactin. Blotting with pY466 Ab detected cortactin phosphorylation mainly in the sample transfected with Zip-cortactin and Zip-Src. These experiments show that acetylation and phosphorylation of cortactin are mutually exclusive: acetylated cortactin is not phosphorylated and *vice versa* (Fig. 3). Another major finding of our study is that when WT cortactin is expressed in transfected cells, at least some of it is acetylated (Figs. 3 and 4) and therefore predicted to be inactive [30].

To determine whether the competition observed between acetylation and phosphorylation of transfected cortactin also holds for the endogenous protein, we carried out experiments in two cell types. The first was WT and HDAC6-deficient MEFs. HDAC6 is the major deacetylase acting on cortactin [30]. As expected, we found the HDAC6-deficient cells to have a significantly higher basal level of acetylated cortactin than did WT cells, as previously described using siRNA techniques [30]. More importantly, we confirmed in both cell types our finding of a competition between acetylation and tyrosine phosphorylation. We did this in two types of IP experiments, one using the 4F11 MoAb and the other using an Ab against acetyl-cortactin (Fig. 5). In the latter IPs, acetyl-cortactin did not show detectable tyrosine phosphorylation, as assessed by either pY466 cortactin Ab or generic pTyr MoAb (Fig. S4). These two IP experiments were repeated on endogenous cortactin in a second cell type, SYF cells, for which Rsrc cells served as control (Fig. 6). Since SYF and Rsrc cells have different levels of tyrosine-phosphorylated cortactin, we performed IPs with 4F11, pTyr Ab and pY466 Ab. As in WT and HDAC6 deficient MEFs, endogenous tyrosine-phosphorylated cortactin was not acetylated.

A major conclusion of our work is that phosphorylation of cortactin has important repercussions on cell spreading, extending the insights of a previous study showing that cortactin mutants mimicking tyrosine phosphorylation affect focal adhesion turnover [28]. Using the FIT system to control tyrosine phosphorylation of cortactin, we analyzed the effect of phosphorylating cortactin on cell location (Fig. S5) and cell spreading (Fig. 7). We found that phosphorylated and unphosphorylated cortactin expressed through transient transfection has an intracellular distribution similar to that of endogenous protein [12]. More importantly, phosphorylation affects cell spreading: cortactin expression facilitated cell adhesion, while tyrosine phosphorylation inhibited it. This phenotype was more noticeable in Rsrc cells, which express Src, than in SYF cells, which do not contain the major SFKs expressed in fibroblasts (Src, Yes and Fyn). This difference between the cell lines is understandable given that many proteins besides cortactin participate in cell adhesion, and many of them are regulated by Src-mediated phosphorylation [43].

In an effort to understand the molecular mechanism underlying the inhibitory effect of tyrosine phosphorylated cortactin on cell spreading, we hypothesized that this post-translational modification would affect the binding of cortactin SH3 domain to interacting proteins that function in cell spreading. One obvious candidate was FAK [37]. Our results in the present study demonstrate that *in vivo*, as previously proposed *in vitro* [10], tyrosine phosphorylation of cortactin prevents the SH3 domain from interacting with FAK and potentially other proteins as well.

While we were preparing this manuscript for submission, researchers reported that a tyrosine phosphorylation-mimicking mutant of cortactin no longer binds FAK and promotes cell motility [44]. This result is comparable to our results obtained with cortactin mutants and with the endogenous protein after *Helicobacter* infection [37]. In the present study, we used not mutant forms of cortactin but the phosphorylated form of the WT protein to demonstrate directly that phosphorylation inhibits cortactin binding to FAK and cell spreading. Our results point to a significant role for tyrosine phosphorylation of cortactin in regulating cell adhesion to fibronectin. This further suggests the possibility that cortactin and its phosphorylation contribute to integrin signaling.

### Model

**Figure 10:**
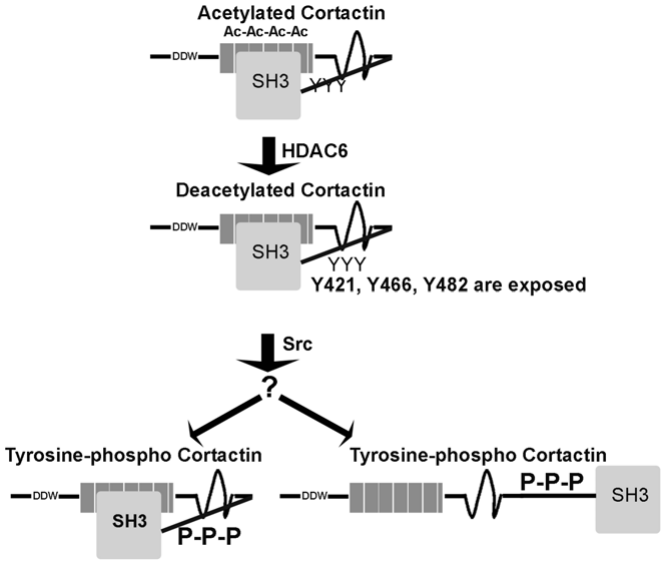
Model for sequential activation of cortactin by deacetylation and phosphorylation. Acetylated cortactin is inactive and probably has a closed conformation that masks the tyrosines targeted by Src. Upon appropriate stimulation, cortactin is deacetylated by HDAC6, exposing the tyrosines, which are then rapidly phosphorylated by Src. This phosphorylation keeps cortactin deacetylated. Whether this tyrosine-phosphorylated cortactin has an open or closed configuration is unknown (question mark).

We propose a model for the ‘sequential’ activation of cortactin (Fig. 10). The major tyrosines targeted by Src are located in the proline-rich region at the C-terminus of the protein. Cortactin has a closed, globular conformation, achieved mainly through interactions among the SH3 domain, the ABR and helical region [27]. This agrees with previous studies showing that in unmodified cortactin, the SH3 domain is masked [10], [22]. Since acetylated cortactin has also been proposed to be inactive [30], we hypothesize that acetylated cortactin has a closed conformation as well.

Based on our observation that acetylation and tyrosine phosphorylation are not present simultaneously, we propose that in acetylated cortactin, the tyrosines targeted by Src are hidden. Analysis of the tertiary structure of cortactin suggests that both acetyl and phosphate groups can be close to each other in space [45], which may explain why one process excludes the other. Acetylation of the ε-amino group of lysines has already been suggested to “rival” phosphorylation in some cases [46]. Some examples of phosphorylation-acetylation switches in the regulation of proteins are already known. For example, Signal Transducers and Activators of Transcription 1 (STAT1) is activated by phosphorylation and inactivated by acetylation [47]. We further propose that upon appropriate stimulation, such as focal adhesion formation during cell spreading, cortactin is deacetylated, mainly by HDAC6, which like cortactin can translocate to the cell periphery [48]. This deacetylated status would be maintained by rapid Src-mediated tyrosine phosphorylation, although we cannot exclude the possibility that other post-translational modifications contribute to inhibiting reacetylation. In essence, we propose that tyrosine-phosphorylated cortactin is a ‘pre-activation state’. At the present moment we do not know whether this species has an open or closed configuration; this will require high-resolution structural analysis.

## Supporting Information

Figure S1 **Western-blotting controls for the transfections of the FIT vectors.** (**A**) To detect our transfected protein, lysates were analyzed by WB with a rabbit cortactin MoAb and a mouse myc MoAb. Both MoAbs recognize transfected cortactin. (**B**) Transfection and cell phenotype controls were performed by WB with HA Ab (in green), and myc and Src MoAbs (in red).
(TIF)

Figure S2 **Specificity of the FIT system as detected with phosphotyrosine generic antibodies.** SYF and Rsrc cells were transfected with different combinations of Src and cortactin FIT fusion vectors (lanes 1–8) or left untransfected (lane 9). The cell lysates were blotted for actin as a loading control, and with a mixture of two generic phosphotyrosine MoAbs: 4G10 and PY20 (Platinum). The major tyrosine-phosphorylated band observed in our lysates corresponded to cortactin detected with rabbit cortactin MoAb (in red) in the lysates cotransfected with ZipA-HA-ΔSrc and ZipB-MycCortactin (lane 5, asterisks).
(TIF)

Figure S3 **Analysis of acetylation and tyrosine phosphorylation of transfected cortactin.** (**A**) Lysates from various transfection combinations (lanes 1–4), treated or not with the deacetylase inhibitor Trichostatin A (TSA), were blotted using pY466 cortactin Ab (pY466) (in green) and 4F11 MoAb (in red) to analyze the phosphorylation of transfected cortactin. (**B**) TSA-treated cell lysates from various transfection combinations (lanes 1–3) were subjected to IP experiments with the pY466 Ab or isotype control Ab (Ctrl.). The IPs were blotted first with acetyl-cortactin Ab, and second with the cortactin 4F11 MoAb; then the membrane was stripped and reprobed with pY466 Ab and with cortactin 4F11 MoAb. The asterisk denotes nonspecific bands.
(TIF)

Figure S4 **Analysis of acetylation and tyrosine phosphorylation of endogenous cortactin in WT and HDAC6-deficient MEFs.** Immunoprecipitates obtained with acetyl-cortactin Ab were blotted with phospho-tyrosine generic mouse MoAb (pTyr) and cortactin rabbit MoAb. There was not phosphorylation signal to coincide with acetylated cortactin.
(TIF)

Figure S5 **Localization of tyrosine-phosphorylated cortactin.** SYF and Rsrc cells were transfected with empty vectors (not shown), with ZipB-MycCortactin and empty vector (TF2) or with ZipB-MycCortactin and ZipA-HAΔSrc (TF3). Cells were fixed and visualized by immunofluorescence using myc MoAb (in blue), pY466 cortactin Ab (in green) and TRITC-phalloidin to label actin cytoskeleton (in red). Pictures were taken on a confocal microscope at 600× magnification. Images were merged and a zoomed view was generated using Leica software. Scale bars are shown. Some cells showed clusters of actin and phospho-cortactin (arrows).
(TIF)
