# Supplementary figures and images for: Cortactin Tyrosine Phosphorylation Promotes Its Deacetylation and Inhibits Cell Spreading
Source: PLoS One. 2012 Mar 30;7(3):e33662. doi: 10.1371/journal.pone.0033662 (PMC3316595; doi:10.1371/journal.pone.0033662)

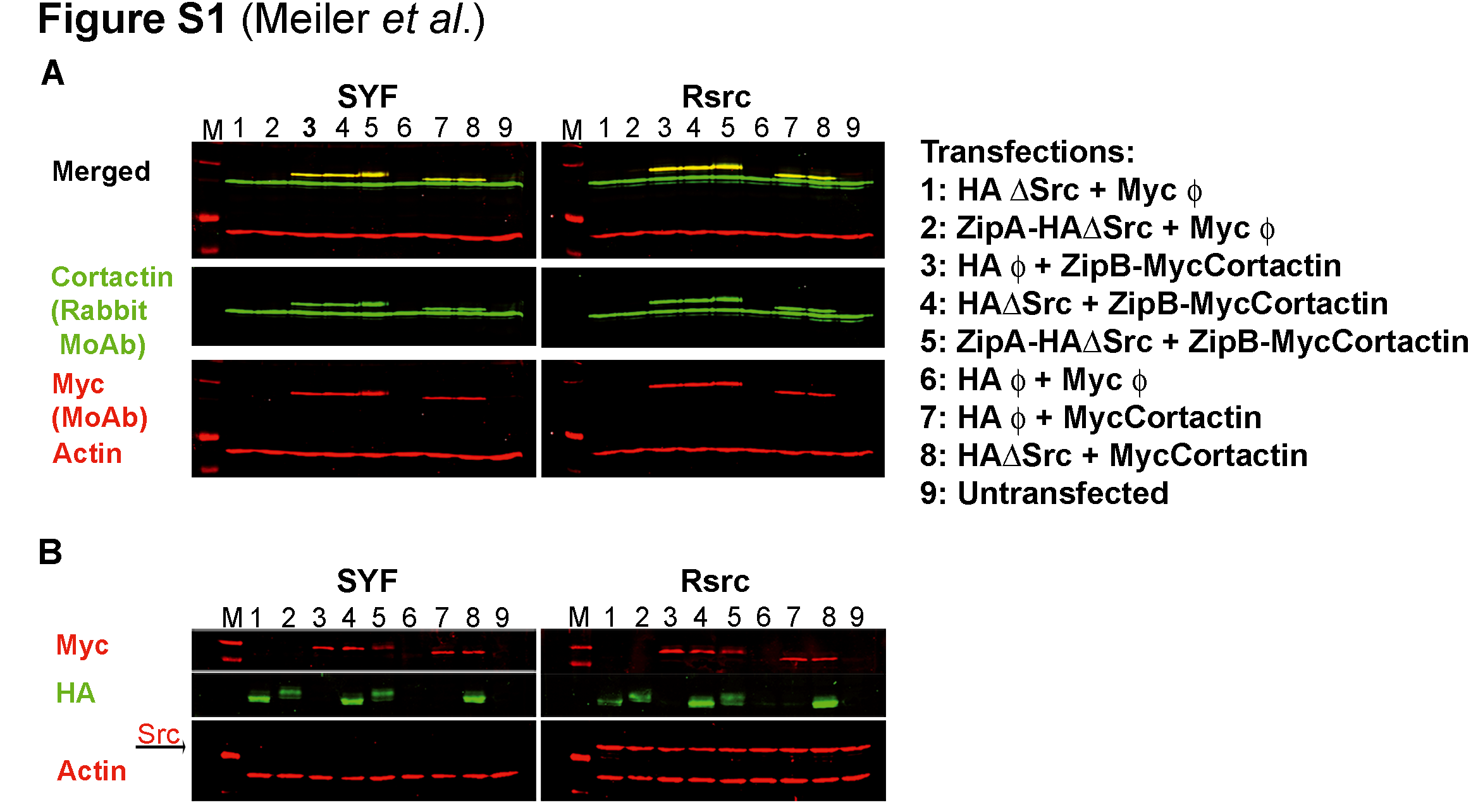

Supplement: Figure S1 — Western-blotting controls for the transfections of the FIT vectors. (A) To detect our transfected protein, lysates were analyzed by WB with a rabbit cortactin MoAb and a mouse myc MoAb. Both MoAbs recognize transfected cortactin. (B) Transfection and cell phenotype controls were performed by WB with HA Ab (in green), and myc and Src MoAbs (in red). (TIF) [file pone.0033662.s001.tif]

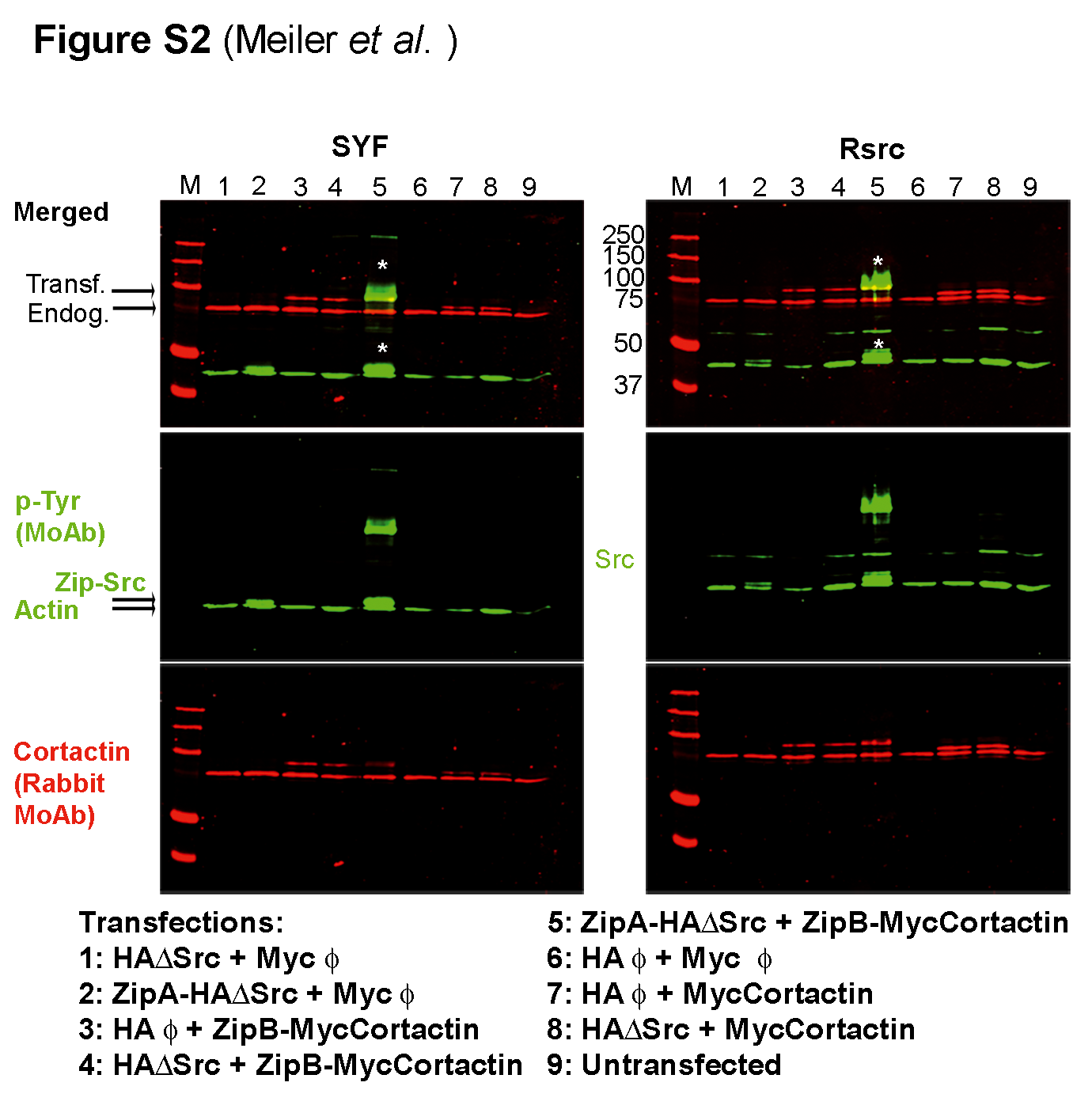

Supplement: Figure S2 — Specificity of the FIT system as detected with phosphotyrosine generic antibodies. SYF and Rsrc cells were transfected with different combinations of Src and cortactin FIT fusion vectors (lanes 1–8) or left untransfected (lane 9). The cell lysates were blotted for actin as a loading control, and with a mixture of two generic phosphotyrosine MoAbs: 4G10 and PY20 (Platinum). The major tyrosine-phosphorylated band observed in our lysates corresponded to cortactin detected with rabbit cortactin MoAb (in red) in the lysates cotransfected with ZipA-HA-ΔSrc and ZipB-MycCortactin (lane 5, asterisks). (TIF) [file pone.0033662.s002.tif]

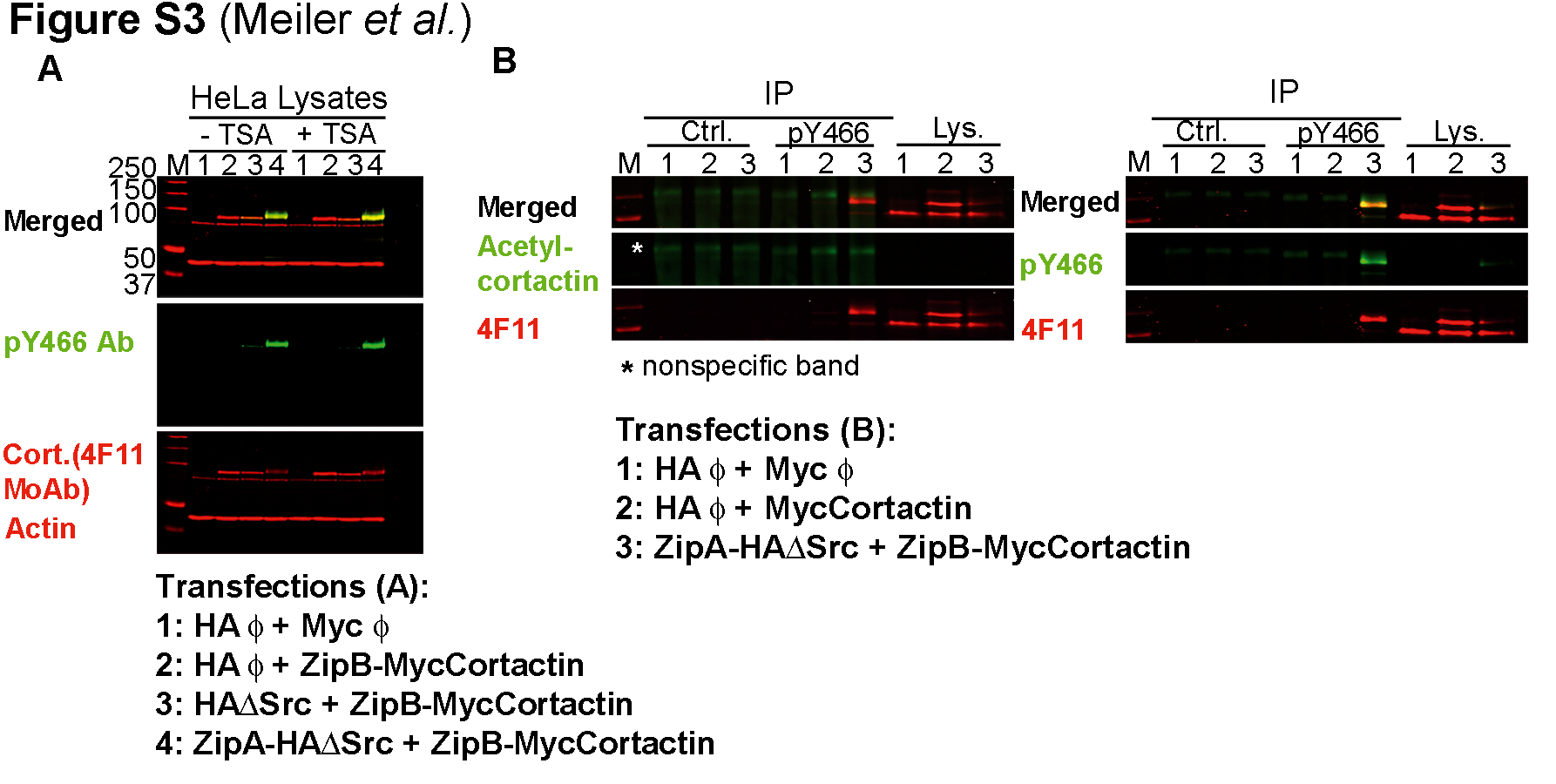

Supplement: Figure S3 — Analysis of acetylation and tyrosine phosphorylation of transfected cortactin. (A) Lysates from various transfection combinations (lanes 1–4), treated or not with the deacetylase inhibitor Trichostatin A (TSA), were blotted using pY466 cortactin Ab (pY466) (in green) and 4F11 MoAb (in red) to analyze the phosphorylation of transfected cortactin. (B) TSA-treated cell lysates from various transfection combinations (lanes 1–3) were subjected to IP experiments with the pY466 Ab or isotype control Ab (Ctrl.). The IPs were blotted first with acetyl-cortactin Ab, and second with the cortactin 4F11 MoAb; then the membrane was stripped and reprobed with pY466 Ab and with cortactin 4F11 MoAb. The asterisk denotes nonspecific bands. (TIF) [file pone.0033662.s003.tif]

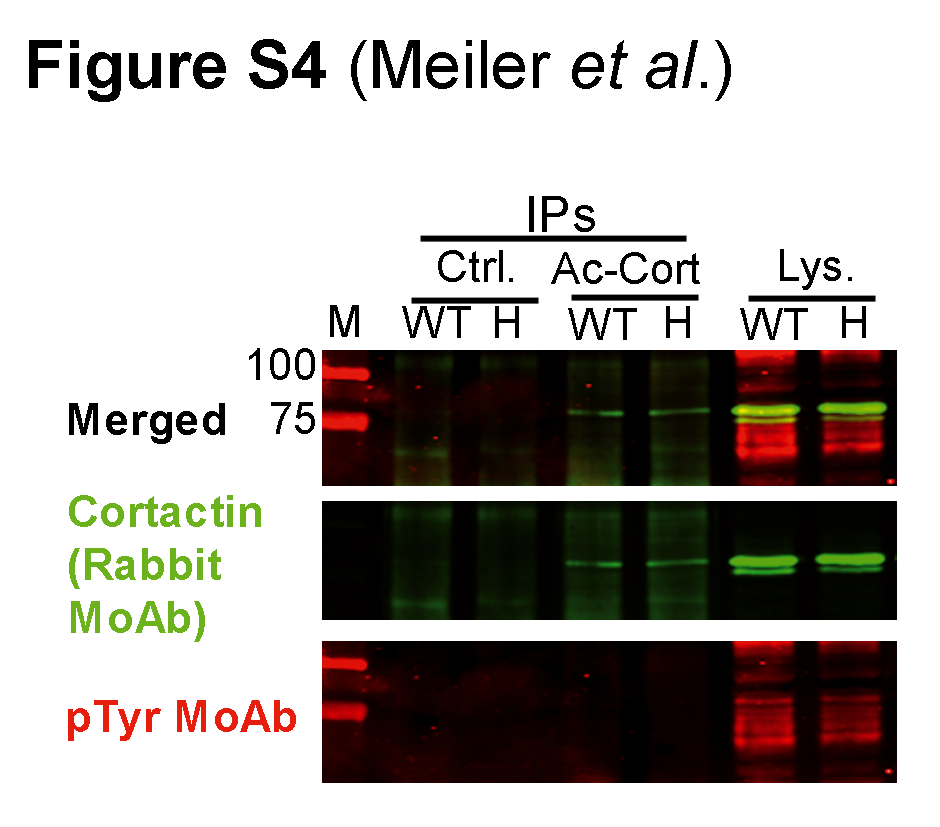

Supplement: Figure S4 — Analysis of acetylation and tyrosine phosphorylation of endogenous cortactin in WT and HDAC6-deficient MEFs. Immunoprecipitates obtained with acetyl-cortactin Ab were blotted with phospho-tyrosine generic mouse MoAb (pTyr) and cortactin rabbit MoAb. There was not phosphorylation signal to coincide with acetylated cortactin. (TIF) [file pone.0033662.s004.tif]

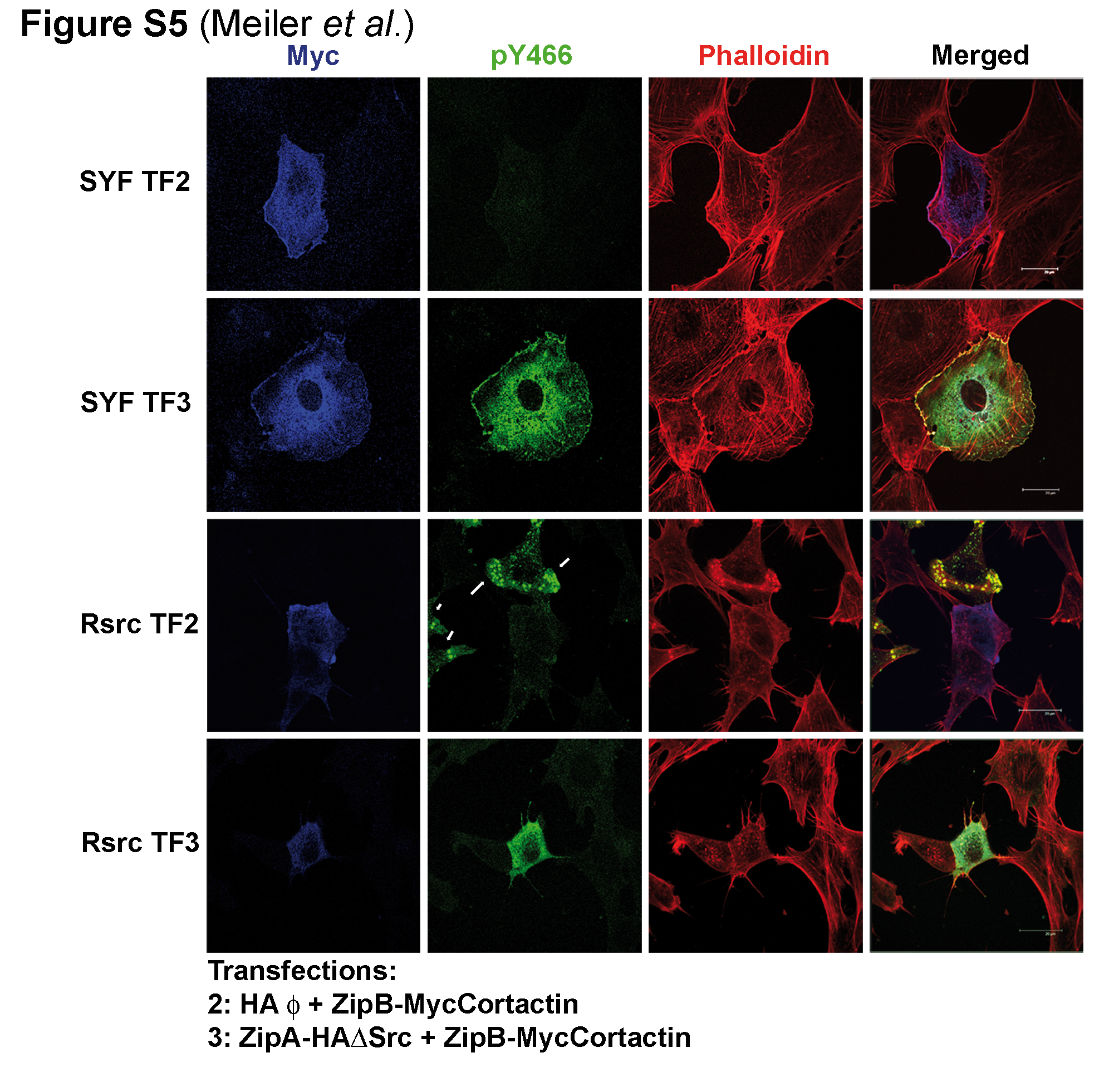

Supplement: Figure S5 — Localization of tyrosine-phosphorylated cortactin. SYF and Rsrc cells were transfected with empty vectors (not shown), with ZipB-MycCortactin and empty vector (TF2) or with ZipB-MycCortactin and ZipA-HAΔSrc (TF3). Cells were fixed and visualized by immunofluorescence using myc MoAb (in blue), pY466 cortactin Ab (in green) and TRITC-phalloidin to label actin cytoskeleton (in red). Pictures were taken on a confocal microscope at 600× magnification. Images were merged and a zoomed view was generated using Leica software. Scale bars are shown. Some cells showed clusters of actin and phospho-cortactin (arrows). (TIF) [file pone.0033662.s005.tif]
